# Scrotal Swelling as a Complication of Hydrochlorothiazide Induced Acute Pancreatitis

**DOI:** 10.1155/2015/265273

**Published:** 2015-06-24

**Authors:** Ivan Nikiforov, Qurat Mansoora, Hassan Al-Khalisy, Sarah Joseph, Pramil Cheriyath

**Affiliations:** Pinnacle Health Systems, 111 South Front Street, Harrisburg, PA 17101, USA

## Abstract

*Background*. Scrotal swelling is a rare complication of acute pancreatitis with few reported cases in the literature. In this case report, we present a 59-year-old male with hydrochlorothiazide induced pancreatitis who developed scrotal swelling. *Case Presentation*. A 59-year-old male presented to the emergency department with sharp epigastric abdominal pain that radiated to the back and chest. On physical examination, he had abdominal tenderness and distention with hypoactive bowel sounds. Computed tomography (CT) scan of the abdomen showed acute pancreatitis. The patient's condition deteriorated and he was admitted to the intensive care unit (ICU). After he improved and was transferred out of the ICU, the patient developed swelling of the scrotum and penis. Ultrasound (US) of the scrotum showed large hydrocele bilaterally with no varicoceles or testicular masses. Good blood flow was observed for both testicles. The swelling diminished over the next eight days with the addition of Lasix and the patient was discharged home in stable condition. *Conclusion*. Scrotal swelling is a rare complication of acute pancreatitis. It usually resolves spontaneously with conservative medical management such as diuretics and elevation of the legs.

## 1. Background

Pancreatitis is an acute medical condition which most commonly occurs due to alcoholism and biliary tract disease in developed countries. It has slowly been increasing in incidence in the United States over the last few decades. Common complications include infection, necrosis, hemorrhage, and pseudocyst formation. An extremely rare complication of pancreatitis is scrotal swelling. An extensive literature review found few of such case reports. In this case report, we present a 59-year-old male with hydrochlorothiazide induced pancreatitis who developed scrotal and penile swelling nine days after the initial presentation.

## 2. Case Presentation

**Figure 1:**
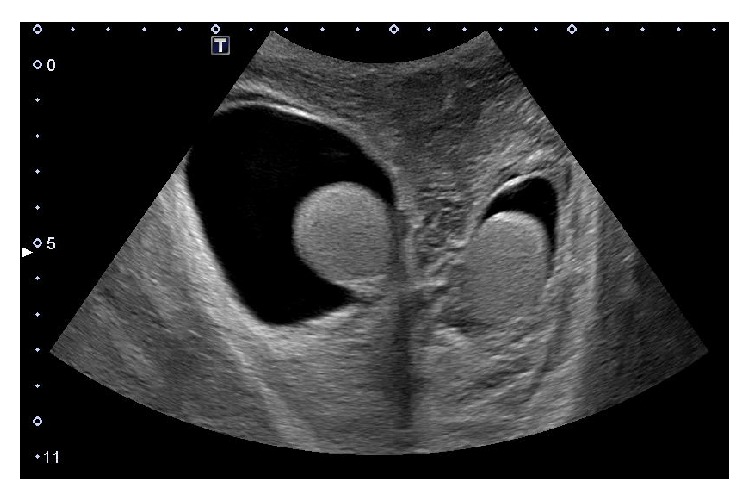


A 59-year-old male with a history of recently diagnosed hypertension presented to the emergency department with sharp epigastric abdominal pain that radiated to the back and chest associated with nausea and nonbloody vomiting. He was started on hydrochlorothiazide (HCTZ) by his primary care physician ten days previously. The patient denied fever, chills, diarrhea, and dysuria. Past medical history was significant for occasional alcohol use. On physical examination, the patient showed abdominal tenderness in the upper quadrants and hypoactive bowel sounds. Vital signs were temperature 36°C, blood pressure 130/80 mm Hg, heart rate 127 beats per minute (bpm), respiratory rate 18 breaths per minute, and oxygen saturation of 99%. White blood cell (WBC) count was elevated at 16.1 × 10^3^ cells/*μ*L with 80 percent neutrophils, lipase 6312 U/L, glucose 178 mg/dL, alkaline phosphatase 69 U/L, alkaline transaminase 84 U/L, albumin 4.5 gm/dL, blood urea nitrogen 18 gm/dL, creatinine 1.32 mg/dL, and hemoglobin 16.1 g/dL. The patient underwent a computed tomography (CT) scan which showed peripancreatic edema and mild pancreatic fluid which was consistent with pancreatitis. The patient was put on a clear diet with aggressive intravenous (IV) fluid hydration. Blood cultures were negative. It was determined that his condition was most likely induced by HCTZ, and the medication was discontinued. His mental status deteriorated and he was transferred to the intensive care unit (ICU). A repeat abdominal CT scan done on the third day of his hospital stay showed increased ascites and peripancreatic edema with necrosis of the pancreatic head. The patient was placed on IV vancomycin and meropenem. The WBC increased to 25.7 × 10^3^ cells/*μ*L. By the sixth day, the patient's condition improved. A third CT scan of the abdomen was done on the eighth day of the hospital stay and showed a decrease in peripancreatic ascites from the previous CT scan and the appearance of peripancreatic fat stranding. On the ninth day of his hospital stay, the patient developed swelling of the scrotum and penis. An ultrasound done the next day showed that both testes were within normal limits and there was a large hydrocele bilaterally (Figure 1). There were no varicoceles or testicular masses. Good blood flow was observed for both testicles. Oral Lasix 40 milligrams twice daily was added to the medications to manage the swelling. Urinalysis was unremarkable. Echocardiogram showed normal ejection fraction with no signs of congestive heart failure. Over the next eight days, the hydrocele and swelling gradually diminished. On the eighteenth day, the patient was discharged home in stable condition.

## 3. Discussion

Typical complications of acute pancreatitis include intra-abdominal infection, pseudocyst formation, pancreatic necrosis, and hemorrhage. Rare complications of acute pancreatitis include scrotal swelling and renal dysfunction. This is the first reported case of scrotal swelling after HCTZ induced acute pancreatitis. To understand the pathophysiology in scrotal swelling due to pancreatic necrosis, it is important to know the relevant anatomy and embryology. The scrotum is composed of the following layers from the inside out: skin, dartos fascia and muscle, external spermatic fascia which is derived from external oblique aponeurosis, cremasteric fascia which is derived from the internal oblique fascia, internal spermatic fascia which is derived from the transversalis fascia, tunica vaginalis, and tunica albuginea. Pancreatic fluid can accumulate and cause erosion structure as far as the groin [1]. Fat necrosis of lower abdominal structures such as external oblique aponeurosis, internal oblique fascia, and transversalis fascia could result in an opening between the abdomen and the scrotum leading to the formation of a hydrocele. An abdominal CT can sometimes show the presence of fluid in the retroperitoneal space, following the ipsilateral paracolic gutter towards the pelvic floor, which can indicate the pancreatic source of the hydrocele. If needed, scrotal aspiration can confirm the diagnosis with the presence of amylase and lipase in abnormal quantities in the effusion, although surgical intervention is typically avoided due to poor wound healing and can deteriorate the patient's quality of life [2, 3].

The most common presentation is painless scrotal swelling without any signs of cellulitis. It is important to differentiate scrotal swelling from testicular torsion, epididymitis and/or orchitis, ventral/inguinal hernia, testicular vasculitis or hemorrhage, and testicular tumor [1, 4]. It is important to rule out testicular torsion if scrotal pain is present since this is a medical emergency. The following physical examination signs increase the suspicion of testicular torsion: elevating the affected testicle which does not relieve the pain (negative Prehn's sign), the cremasteric reflex being absent (positive Rabinowitz' sign), and a focal, dark discoloration of the scrotum (Blue Dot Sign) [4]. A color duplex ultrasound can rule out testicular torsion and indirect bowel herniation or strangulation through the inguinal canal. Additionally, it will detect testicular masses if testicular cancer is suspected. When epididymitis is suspected, additional labs such as urine analysis, culture, and screening for sexually transmitted infections can be ordered. Overall, little is known about the correlation between pancreatitis and scrotal swelling due to the small number of case reports in the literature.

Scrotal edema, without the presence of an emergent testicular condition, usually subsides alongside the resolution of pancreatitis. Abdominopelvic CT can show the presence of fluid in the retroperitoneal space and can be followed up with ultrasound if additional imaging of the scrotum is needed to rule out urgent medical conditions [1]. Scrotal aspiration can confirm the diagnosis, but surgical intervention is typically avoided due to poor wound healing that can deteriorate the patient's quality of life. Conservative treatment is the method of choice [4, 5]. Diuretics and elevation of the legs can be utilized to manage moderate circumstances. Previous reported cases were managed medically and improved without complications.

## References

[1] Kim S., Je B., Lee S. H., Cha S. H. (2011). Scrotal swelling caused by acute necrotizing pancreatitis: CT diagnosis. *Abdominal Imaging*.

[2] Lee A. D., Abraham D. T., Agarwal S., Perakath B. (2004). The scrotum in pancreatitis: a case report and literature review. *Journal of the Pancreas*.

[3] Lin Y.-L., Lin M.-T., Huang G.-T. (1996). Acute pancreatitis masquerading as testicular torsion. *The American Journal of Emergency Medicine*.

[4] Chen Y.-S., Chiang I.-N., Yang S. S.-D. (2009). An unusual cause of acute scrotum: pancreatitis-related scrotal pain. *JTUA*.

[5] Liu K.-L., Lee T.-C., Wang H.-P. (2006). A tender scrotum and inguinal mass caused by pancreatitis. *Clinical Gastroenterology and Hepatology*.

